# A Neural Network for the Prediction of the Visual Acuity Gained from Vitrectomy and Peeling for Epiretinal Membrane

**DOI:** 10.1016/j.xops.2025.100762

**Published:** 2025-03-13

**Authors:** Rupert Kamnig, Noah Robatsch, Anna Hillenmayer, Denise Vogt, Susanna F. König, Efstathios Vounotrypidis, Armin Wolf, Christian M. Wertheimer

**Affiliations:** Department of Ophthalmology, University Hospital Ulm, Ulm, Germany

**Keywords:** Deep learning, Epiretinal membrane, Membrane peeling, Pars plana vitrectomy, Retinal imaging

## Abstract

**Purpose:**

A significant proportion of patients with epiretinal membrane (ERM) demonstrate improvement in visual acuity (VA) 3 months after pars plana vitrectomy (PPV) and membrane peeling. The identification of these patients before surgery is clinically relevant.

**Design:**

This retrospective study was conducted to establish a neural network to predict improvement using preoperative clinical factors and OCT.

**Subjects:**

A total of 427 eyes from 423 patients who underwent a PPV for primary idiopathic ERM combined with or without cataract surgery were included.

**Methods:**

The data were automatically labeled according to whether an improvement of at least 2 logarithm of the minimum angle of resolution lines was observed. A multilayer perceptron was trained using a set of 7 clinical factors. The images were processed using a convolutional network. The output of both networks was concatenated and presented to a second multilayer perceptron. The dataset was divided into training, validation, and test datasets.

**Main Outcome Measures:**

The accuracy of the neural network on an independent test dataset for the prediction of postoperative VA was analyzed. The impact of individual clinical factors and images on performance was assessed using ablation studies and class activation maps.

**Results:**

The clinical factors alone demonstrated the highest accuracy of 0.74, with a sensitivity of 0.82 and a specificity of 0.67. These results were obtained after the exclusion of less significant factors in an ablation study. The inclusion of the factors age, preoperative lens status, preoperative VA, and the distinction between combined phacovitrectomy and vitrectomy yielded the most accurate results. In contrast, the use of ResNet18 as a neural network for image processing alone (0.61) or images combined with clinical factors (0.70) resulted in reduced accuracy. In the class activation map, image regions corresponding to the outer, central, and inner retina appeared to be important for the decision-making process.

**Conclusions:**

Our neural network has yielded favorable results in predicting improvement in VA in approximately 3-quarters of patients. This artificial intelligence–based personalized therapeutic strategy has the potential to aid decision-making. Future studies are to assess the clinical potential and generalizability and improve accuracy by including a more extensive dataset.

**Financial Disclosure(s):**

The author(s) have no proprietary or commercial interest in any materials discussed in this article.

Epiretinal membranes (ERMs) present with symptoms including metamorphopsia, visual field loss in microperimetry, reduced contrast sensitivity, aniseikonia, or even reduction in vision-related quality of life.^1^ Regarding the prevalence, large epidemiological studies have reported that 17% to 34% of the general population has detectable ERM in OCT.^2^^,^^3^ In a small proportion of these patients, if symptoms are significant, surgical removal of the ERM by pars plana vitrectomy (PPV) with ERM and internal limiting membrane peeling is the only available treatment. The functional outcomes of this procedure are generally favorable with a reduction in metamorphopsia and improvement in visual acuity (VA) in the majority of cases.^1^^,^^4^

However, despite advancements in surgical techniques and the low incidence of surgical complication, approximately 30% to 50% of patients do not improve in VA of >2 lines after surgery.^1^^,^^5^^,^^6^ Identification of patients at risk of nonimprovement is necessary to determine which patients would benefit from surgical intervention, which is also an integral part of preoperative patient counseling. In this context, a range of preoperative clinical and morphological OCT biomarkers have been identified as prognostic factors to predict visual outcomes after surgery.^5^ To date, the age of onset, preoperative VA, duration of symptoms, as well as assessment of outer retinal segments, intraretinal cystoid spaces, and inner retinal layers have been found to be of value. Clinically, the decision to proceed with surgical intervention is based on the surgeon's expertise and experience in evaluating the multifactorial prognostic indicators. To assist the surgeon, neural networks offer the potential to extract meaningful insights from complex multifactorial datasets and recent studies suggest that deep learning may be a valuable tool for predicting postoperative outcomes in the future.^7^^,^^8^

The aim of this study was to develop a neural network capable of predicting the individual risk of nonimprovement of VA after PPV and peeling for primary ERM based on a comprehensive set of preoperative clinical factors in combination with preoperative OCT images.

## Methods

### Study Design

A total of 427 eyes from 423 patients who underwent a PPV for primary idiopathic ERM stage 2 to 4^9^ were included in this retrospective study and treated between January 2020 and October 2022 at the Department of Ophthalmology at the University of Ulm in Germany. The study was approved by the Ethics Committee of the University of Ulm (ethics approval ID: 332/22) and conducted in accordance with the tenets of the Declaration of Helsinki. Informed consent was not obtained because the study was retrospective with data from routine observation.

### Inclusion Criterion

The decision to proceed with surgical intervention was made in cases where there was evidence of progressive VA impairment and/or metamorphopsia, in addition to a loss of foveal depression due to ERM. In cases where a significant cataract was present, a combined phacovitrectomy was performed. Consequently, the study included patients who met the following criteria: (1) a diagnosis of idiopathic ERM stage 2 to 4, (2) preoperative macular OCT, (3) an uneventful PPV and membrane peeling, which was defined as no complications or abnormalities noted in the surgeon's report other than peripheral breaks, and (4) reliable 3-month follow-up with preoperative and postoperative best-corrected visual acuity (BCVA) as determined using an automated refractor. Patients with secondary ERM were excluded.

### Surgical Procedure

A team of 4 experienced vitreoretinal surgeons (A.W., E.V., Melih Parlak, and Hans-Jürgen Buchwald) performed small-gauge vitrectomy^10^, ^11^, ^12^ under retrobulbar or general anesthesia. Following the completion of the core vitrectomy, the posterior vitreous detachment was performed. The peeling of the epiretinal tissue and internal limiting membrane was performed using Eckardt end-gripping microforceps, with the assistance of brilliant blue staining (DORC). In the event of a retinal break, vitreous base shaving and retinal endolaser were conducted. Air was utilized for tamponade. In cases where a diagnosis of concomitant cataract was established, standard microincisional cataract surgery was performed, which included phacoemulsification and intraocular lens implantation into the capsular bag.

### Neural Network Input Data

From the electronic medical records, we retrospectively collected 7 preoperative clinical factors: (1) sex, (2) age, (3) intraocular pressure (IOP), (4) preoperative BCVA, (5) preoperative lens status, (6) type of surgery, and (7) postoperative BCVA. At baseline, all eyes underwent spectral-domain OCT using one of 2 platforms: Spectralis (Heidelberg Engineering GmbH, Heidelberg, Germany) or the Zeiss Cirrus 5000 (Zeiss Meditec, Jena, Germany). The most central section B-scan through the fovea was manually selected for processing in the neural network.

### Neural Network Output and Labeling

The patients were divided into 2 groups and labeled accordingly. The first group consisted of those patients who exhibited a postoperative VA improvement of at least 2 lines on the logarithm of the minimum angle of resolution (logMAR) chart. The second group comprised patients who did not improve.

### Neural Network Architecture

**Figure 1:**
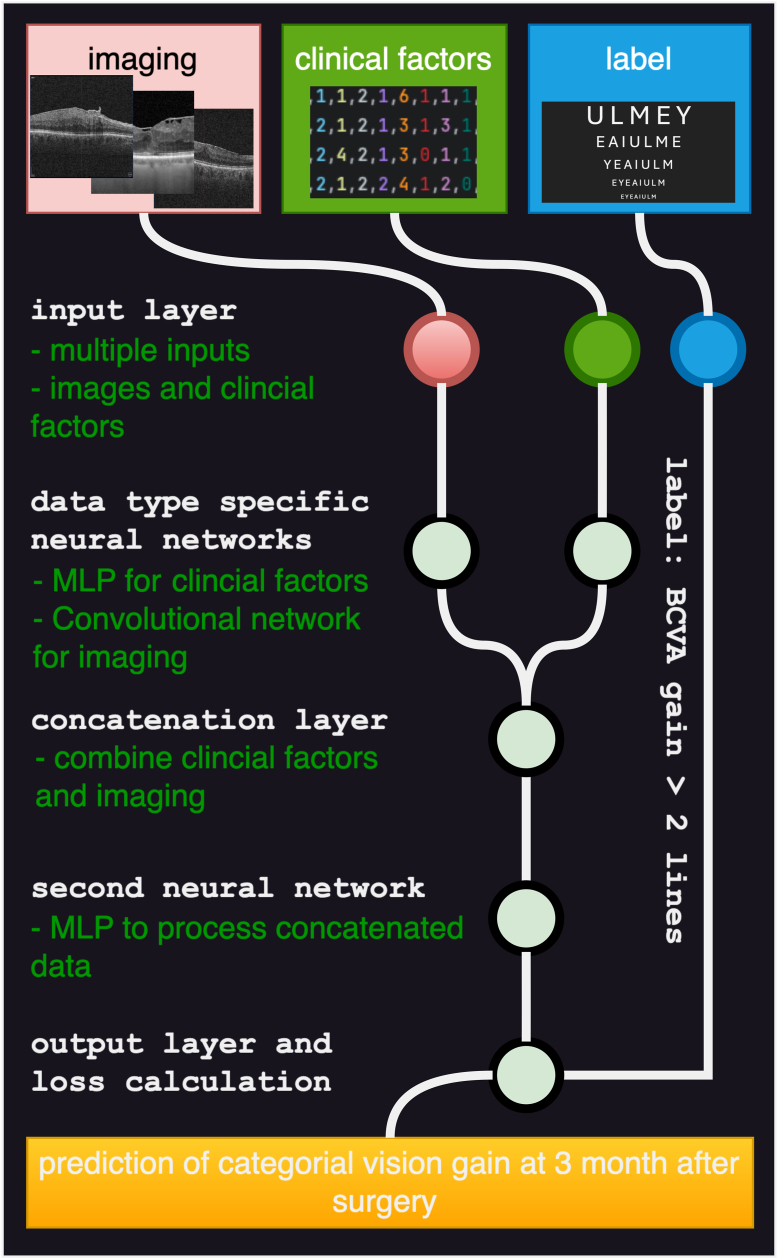
This is an outline of the neural network utilized in this manuscript. The network was constructed to handle 2 different types of input data. Images were processed with a convolutional neural network (ResNet18), and numerical clinical factors were presented to a MLP. Both resulting tensors were concatenated, and a third neural network consisting of a multilayer perceptron computed the output result, which was compared to the label by calculating the binary cross-entropy loss. Back propagation was achieved by adaptive moment estimation. BCVA = best-corrected visual acuity; MLP = multilayer perceptron.

A neural network architecture was used, as described previously.^13^ The programming was performed by CMW using PyTorch (The Linux Foundation) as the machine learning framework and the Pycharm integrated development environment (JetBrains). A neural network can self-learn to produce a desired output when given a set of data and examples of the desired output (labels); the data are processed within the neural network. In this study, the 2 different types of data (clinical factors and OCT images) were each processed in a different neural network. Images were processed in a convolutional neural network (ResNet18) and numerical clinical factors were processed in a multilayer perceptron neural network (1 hidden layer of 14 neurons). When both types of data were processed simultaneously in the neural network, the outputs of the 2 different networks were concatenated and the result was then processed by another multilayer perceptron neural network. The output was a decision as to whether VA improved by 2 lines (see Fig 1). First, the different types of data obtained from the clinical dataset were presented to the neural network, which involves rescaling of the data from the original range and transforming it into tensors that can be processed by the neural network. For this purpose, the PyTorch data loader was used in conjunction with the CSV, NumPy, Pillow, and Pandas libraries to facilitate the transformation of the 2 data types into tensors. Three-channel Portable Network Graphics images of central OCT scans were cropped and normalized according to the ImageNet standard using Torchvision transforms. After processing in the network, for self-learning, the desired output prediction must be compared with the true label obtained from the postoperative VA gain and an error (binary cross-entropy loss) is calculated. The neural network is then optimized according to this error calculation by an algorithm (adaptive moment estimation optimizer). We tried to achieve generalizability by dividing the dataset into 3 part: a training dataset (70% of the patients), a validation dataset (20%), and a test dataset (10%). Training was performed on the training dataset. A reduction in error (loss) in a test of the validation dataset triggered a checkpoint save of the trained network, and training was terminated after a specified number of epochs without a reduction in the loss of the validation dataset (early stopping). Hyperparameters were optimized by systematically iterating through possible combinations of batch sizes (2, 4, 6, 8, 16, 32) and learning rates (0.1–0.000001 in order of magnitude).

### Class Activation Maps

In an effort to enhance the interpretability of the convolutional neural network (ResNet18) utilized, we employed class activation maps, as described elsewhere,^14^ which quantify the activation of the learned weight parameters in the final layer of a convolutional neural network according to the class selected. Subsequently, these activation maps are rendered visually, and the results correlated to the location in the original image. This approach emphasizes the regions of the image that exhibit the most significant activation, thereby highlighting the spatial patterns often associated with the image features utilized by the neural network for its decision-making process.

### Ablation Study

To assess the importance and role of the 7 different clinical factors in the neural network's decision-making process, an ablation study was conducted, as described elsewhere.^15^ To this end, single clinical factors were selectively removed from neural network training and testing. The different performance metrics (accuracy) with and without these factors included were then compared to determine the contribution of the single parameters. This process was repeated for each of the 127 possible combinations of the 7 clinical factors, with each combination evaluated independently.

### Network Performance Metrics

The performance of our multiple data inputs in forecasting the individual risk of VA improvement was evaluated through the calculation of confusion matrix metrics, including accuracy, sensitivity, and specificity, all derived from the withheld test dataset.

### Statistical Analysis

All computations and data collection were performed in the PyCharm integrated development environment and in Python 3.12, using the following libraries: CSV, RegEx, Pandas, and NumPy. The graphs were plotted using either matplotlib.pyplot or GraphPad PRISM9 (GraphPad Software, Inc). A 2-sample *t* test was performed using GraphPad PRISM9 to compare the 2 groups. For categorical comparisons, the Pearson chi-square test was calculated using SPSS28 (IBM). A *P* value of 0.05 was considered statistically significant.

## Results

### Demographics

Of the 427 eyes that underwent PPV for primary idiopathic ERM at the 3-month follow-up, 215 (50%) showed an improvement of 2 or more lines, while 212 (50%) did not achieve a 2-line improvement in VA. The mean age of all patients was 71 ± 8 years, and the mean preoperative VA was 0.5 ± 0.3 logMAR. When comparing the 2 groups, the mean age was found to be statistically significantly younger in the patients who exhibited improvement (*P* = 0.046). In addition, preoperative VA was found to be a significant predictor of postoperative VA outcome, with patients who demonstrated improvement having worse preoperative VA (*P* = 0.0001). There were no statistically significant differences observed in biological sex, laterality, lens status, or type of surgery (phacovitrectomy) and IOP between the 2 groups (Table 1).

**Table 1:** Statistical Comparison of Preoperative Clinical Factors Revealed That Patients Who Showed Improvement of Visual Acuity Were Younger and Had Worse Preoperative Visual Acuity

	Visual Acuity Improved	Visual Acuity Not Improved	*P* Value
n (eyes)	215	212	
Age (years)	70 ± 9	72 ± 8	**0.046**
Biological sex (male)	127 (59%)	121 (57%)	0.7
Laterality (right eyes)	111 (52%)	106 (50%)	0.7
Lens status (phakic eyes)	147 (68%)	128 (60%)	0.1
Surgery (phacovitrectomy)	133 (62%)	117 (55%)	0.2
Preoperative visual acuity (logMAR)	0.63 ± 0.34	0.38 ± 0.21	**0.01**
Intraocular pressure (mean)	14.5	14.7	0.5

logMAR = logarithm of the minimum angle of resolution.

Bold values are statistically significant.

### Neural Network Performance

**Figure 2:**
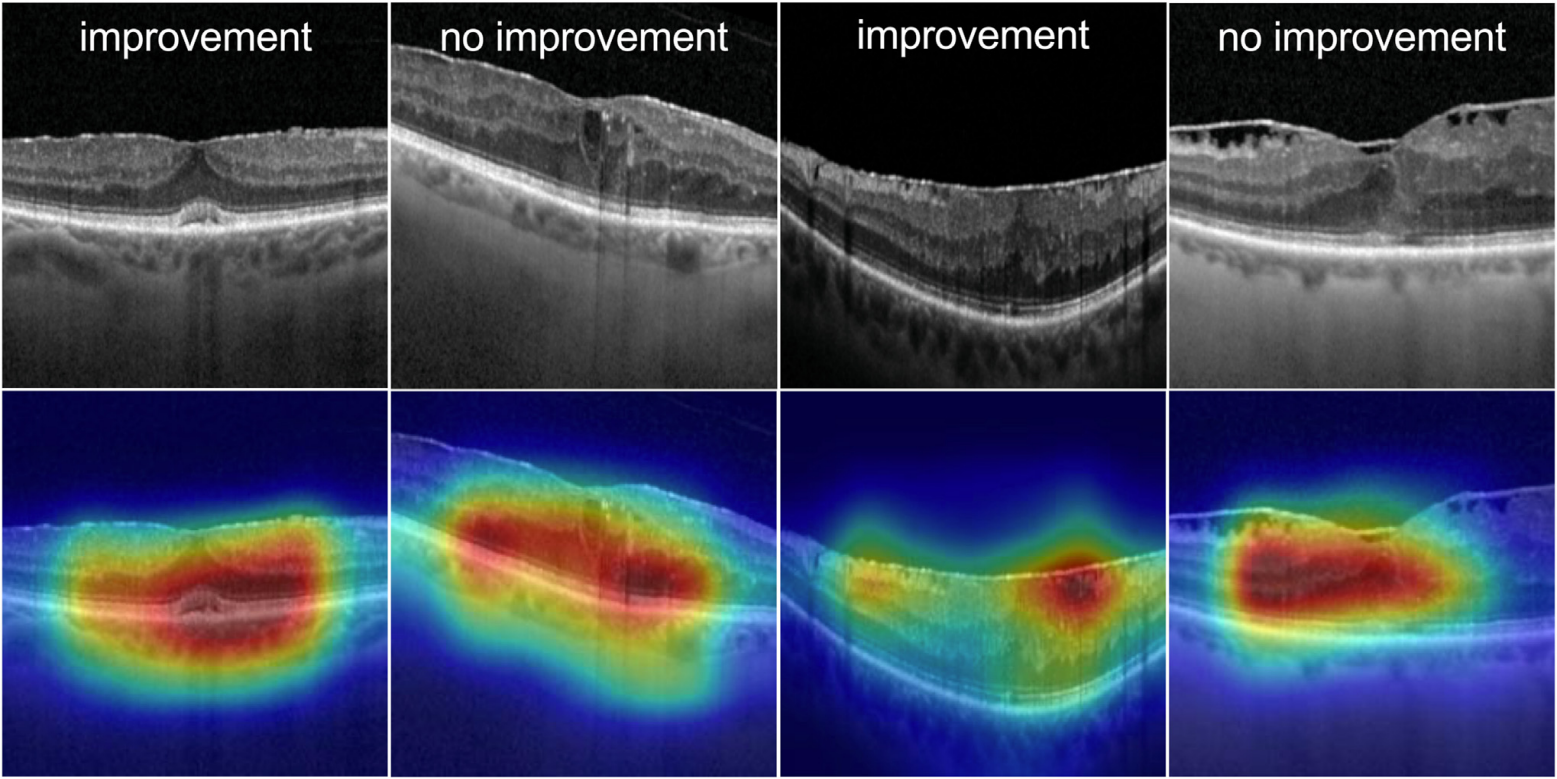
By employing a systematic approach that includes all possible combinations of the 7 numerical clinical factors, it is possible to determine which factors are most influential in determining visual acuity outcomes following vitrectomy and peeling. The various combinations of clinical factors are presented in a matrix plot. A multitude of multilayer perceptrons were trained and tested for their accuracy in the ablation study, with each of the 127 possible combinations being evaluated separately. The y-axis displays the combinations, with each row representing 1 combination, while the x-axis represents the different clinical factors, with each column representing 1 factor (1 = age, 2 = sex, 3 = side, 4 = preoperative lens status, 5 = combined phacovitrectomy or vitrectomy, 6 = preoperative visual acuity, 7 = intraocular pressure). If a clinical factor is excluded from a particular combination, it is shown in black. The results for the maximum accuracy are color-coded for each row, as illustrated in the figure on the left. The inclusion of the following factors provided the highest levels of accuracy: age, preoperative lens status, preoperative visual acuity, and whether combined phacovitrectomy or vitrectomy alone was performed.

To gain insight into the effects of the different clinical factors and the OCT images on the overall performance of the neural network in the decision-making process, the different types of data were presented separately or in combination to the neural network for training and testing. The resulting performance after removal of a component can then be analyzed, and conclusions can be drawn about the contribution of each factor. In this context, when images were utilized as the sole input, the resulting accuracy was relatively low, at 0.61, with a sensitivity of 0.61 and a specificity of 0.81 (Table 2). Upon combining the OCT images with the clinical factors, an improvement in accuracy was observed, reaching a satisfactory level of 0.70, and the specificity and sensitivity reached 0.7 and 0.76, respectively. A comprehensive ablation study of all 127 possible combinations of the clinical factors was performed to ascertain the optimal combination of clinical factors. The results demonstrated that the highest accuracy (0.74) was achieved when clinical factors were used, and the sensitivity and specificity were 0.82 and 0.67, respectively. The inclusion of age, preoperative lens status, preoperative VA, and whether combined phacovitrectomy or vitrectomy was performed seemed to lead to the highest accuracies (Fig 2).

**Table 2:** This Comprehensive Overview of All Data Types Processed by Specific Neural Networks is Presented in Accordance with the Maximum Performance Measurements Documented after Training and Testing

Neuronal Network Design	Accuracy	Sensitivity	Specificity
Clinical factors and images (ResNet18 + MLP)	0.70	0.70	0.76
Clinical factors only (multilayer perceptron)	0.74	0.82	0.67
Images only (ResNet18)	0.61	0.61	0.81

The multilayer perceptron showed the highest accuracy of 0.74 when clinical data were employed and after excluding less significant factors in an ablation study.

### Class Activation Maps

**Figure 3:**
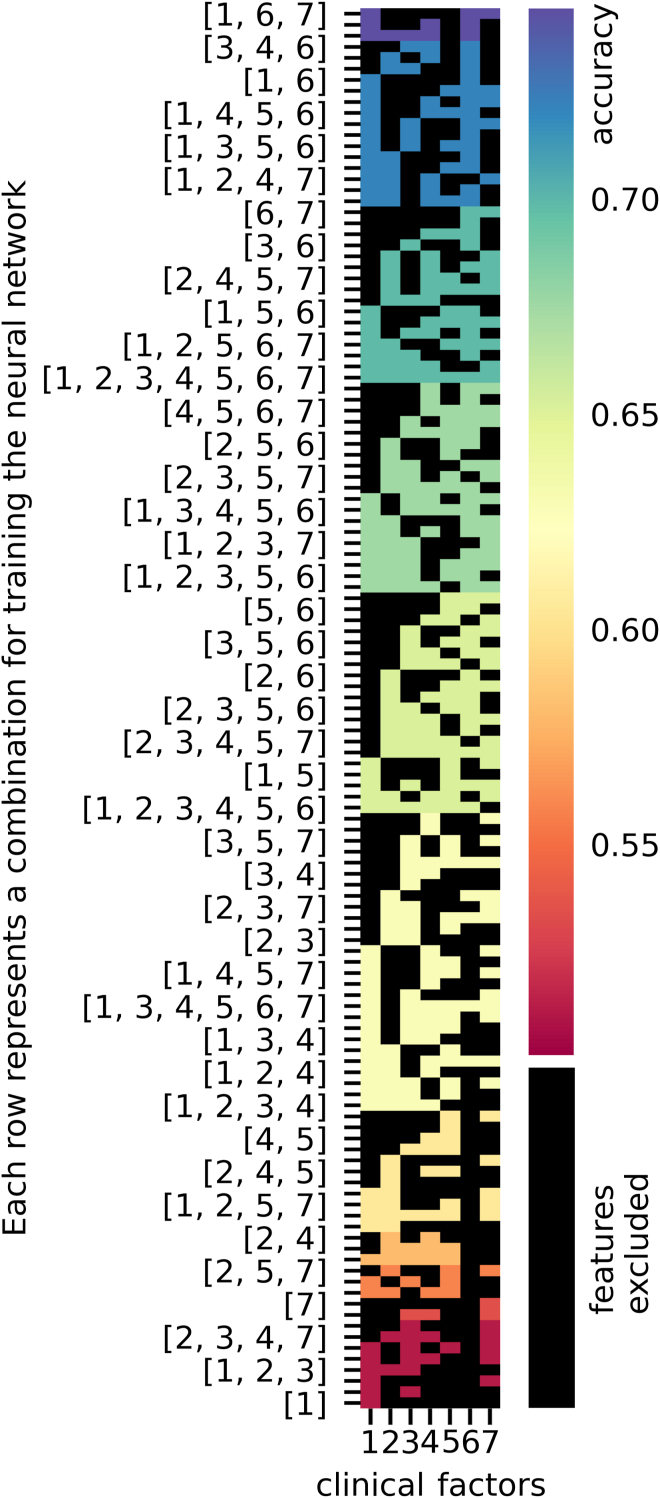
Gradient-weighted class activation maps are obtained from a ResNet18 trained with images only. Other than the detection of central retinal regions, no other patterns or biomarkers could be derived from the class activation maps.

Class activation maps highlight the spatial patterns often associated with the image features used by the neural network for decision-making. This helps to improve the interpretability of the results (Fig 3). The neural network shows activation in areas of the OCT scan representing the central foveal region. Areas of the outer, central, and inner retina appear to be important in discriminating whether VA is improving. Other than the detection of the central regions, we could not derive any other patterns or potential biomarkers from the class activation maps. These results confirm that the model focuses on the same regions of the images for its predictions as would be used by humans for clinical reasoning.

## Discussion

This study shows that artificial intelligence and deep learning have the potential to predict the individual risk of nonimprovement of VA after PPV and peeling for primary ERM based on preoperative clinical data. We were able to formulate a true and personalized visual prognosis for 3-quarters of our patients, with good sensitivity and specificity. In the future, this may help to improve preoperative decision-making and patient counseling, leading to more personalized therapeutic strategies and management. In addition, our study is consistent with the results of previous studies using artificial intelligence to predict the outcome of vitrectomy for ERM.^16^^,^^17^ A methodology similar to our approach has been published, where the imaging and numerical data were integrated into a neural network to predict an increase of more than 2 Snellen lines with similarly favorable outcomes.^8^ Furthermore, it has been shown that the determination of a numerical value of postoperative VA^18^^,^^19^ and postoperative central foveal thickness^7^ for each individual patient by a deep learning regression model is generally possible with a reasonable error rate. This and our results highlight the potential of the proposed artificial intelligence methodology in the given context.

Identifying factors that need to be presented to the neural network to reach high prediction performance and learning properties will facilitate more efficient future applications in this research area. For this purpose, a systematic evaluation was conducted to identify those important factors by systematically excluding them from the training and testing of the neural network and then analyzing the resulting performances. In addition to imaging, the most influential factors were age, preoperative lens status, preoperative VA, and whether combined phacovitrectomy or vitrectomy was performed. These findings align with those of previous studies.^20^, ^21^, ^22^, ^23^ Furthermore, other studies demonstrated that the duration of symptoms and presence of metamorphopsia are additional clinical factors that are predictive of VA.^10^^,^^24^

Other studies indicate that patients exhibit an improvement in VA within the first 3 months after surgery. For example, a cohort of 504 eyes exhibited a gain of ≥2 lines in 42% of patients at 3 months, 34.7% at 6 months, and 48.1% at 12 months following vitrectomy and ERM peeling.^5^ This figure is slightly lower than the 50% observed in our study. We deliberately chose a 3-month follow-up period to increase the number of cases needed to train the network, as longer follow up was associated with higher loss to follow-up. A challenge common to all studies is establishing universal visual expectations for patients following treatment. This is particularly difficult in cases where refraction and other factors such as cataract surgery may have confounded the results. This is underscored by the finding that pseudophakic lens status was associated with superior visual improvement at 3 months in other studies.^5^

We observed that a higher preoperative VA was an indicator for no improvement of VA in the postoperative period, which may be due to a ceiling effect. A VA cutoff as a factor in the decision to perform surgery has been discussed in the literature. A systematic review of multiple studies with 639 eyes found a 0.15 logMAR as the most liberal and a 0.5 logMAR as the most conservative cutoff to perform surgery.^6^ (The mean preoperative VA in this review was 0.55 logMAR, slightly lower than our 0.5 logMAR.) The main finding was that the greatest mean change in VA was observed in studies that used a conservative preoperative VA threshold and demonstrated a ceiling effect for higher VAs.^6^ Future studies will need to reach a consensus of whether to define a mean change in VA, as used in our study, or use the absolute value of VA reached as surgical success. If an absolute value would be chosen, there is a high possibility that a lower preoperative VA would be an indicator of not reaching a certain higher threshold of VA as in ERM surgery VA loss can often not be restored fully by surgery.^7^ Our neural network could be trained with the other label accordingly.

The combination of cataract surgery and PPV with ERM peeling has to be considered as a bias in our study because the causality of VA improvement cannot be attributed to one of the 2 types of surgery.^11^ In addition, we were not able to analyze the difference between the patients who remained phakic in the neural networks because the group number of these patients was too small. In our institution, combined phacovitrectomy is considered the standard of care, and out of a total of 275 phakic eyes, 250 underwent combined phacovitrectomy. Overall, the combined approach has several advantages, including shorter visual rehabilitation time, prevention of future secondary surgery, clearer visualization during surgery, and a favorable cost-benefit ratio.^12^^,^^25^ Statistically, there was no significant difference in the preoperative lens status and the number of phacovitrectomies performed in the improvement group compared to the no improvement group. Both factors were included to help the neural network predict the individual risk of nonimprovement. Future studies with more cases could focus on training 2 separate neural networks for phakic and nonphakic patients to avoid this bias completely.

Class Activation Maps have the ability to enhance the interpretability of convolutional neural networks in computer vision tasks.^14^ They highlight the regions of an image that contribute most to the model's classification decision and provide insight into the decision-making process through heat map visualization. The regions highlighted in our study correspond to those used in clinical decision-making. OCT, as a diagnostic tool for ERMs, offers high sensitivity for diagnosis, characterization, and longitudinal follow-up.^26^ The neural network demonstrated activation in the central fovea, as well as in the outer and inner areas of the retina.^5^ To date, several imaging-based parameters have been identified as predictors of postoperative visual outcome. In patients with ERM, greater foveal thickness,^10^ an intact photoreceptor inner segment, and ellipsoid zone (especially the cone outer segment tips) are associated with a good postoperative visual outcome.^27^^,^^28^ However, our study did not identify a single region of interest other than the detection of central retinal regions from the class activation maps that was determinant for classification. These central regions are consistent with the image features published in previous studies that have identified central photoreceptor disruption and tractional abnormalities in the central foveal bouquet as being associated with poor postoperative VA.^8^^,^^24^^,^^29^ In this instance, the activation observed in the class activation map allows validation that the model focuses on the relevant central location of the images, as it showed good agreement with the location of clinically relevant markers in this area.

Neural networks have been an important area of research in ophthalmic image analysis, demonstrating good performance in the diagnosis and classification of ocular diseases.^30^ The prediction method used in this study is inherently more challenging as a classification task, and it is reasonable to expect a lower level of accuracy.^13^ This is because it uses historical or recent data to predict future outcomes, which may be influenced by a number of unpredictable or unknown factors. In addition, a common problem with the use of deep learning is the lack of generalizability of neural networks trained on a single institution's specific dataset. The generalizability is therefore limited to the patients of that institution, and it is not possible to draw valid conclusions about other cohorts. In this context, our study has to be seen as a general proof of the feasibility of the given approach, and further studies with more cases in a multicenter setting have to be collected to verify our results in other cohorts.

Limitations of the study include its retrospective nature, due to which it was not possible to include confounder-free cases where refraction and optical media were adequately accounted for; however, we attempted to include these factors in our neural network for training. Because of the retrospective nature, 2 different OCT platforms were used, which may result in lower accuracy in image training and testing. In addition, this study used a small number of cases for a deep learning approach due to the relative rarity of the disease. While VA has been shown to improve before metamorphopsia, the follow-up of 3 months may not be sufficiently long to observe further improvements. In this context, our study could serve as a proof of concept that prediction of VA in ERM surgery is feasible with neural networks and preoperative data. Higher decision accuracy is expected with more data and larger neural networks, possibly in a multicenter and prospective design, to avoid bias and gain more cases.

Because there is no consensus on a label for treatment success after ERM peeling, this study focused on VA gain. We did not address other outcomes such as visual field loss on microperimetry, reduced contrast sensitivity, aniseikonia, or even a reduction in vision-related quality of life, which are important factors that may affect individuals affected by this condition. Further investigation into the neural networks that determine the resolution of metamorphopsia represents a promising avenue for future research. A longer follow-up period would be required, as the resolution of metamorphopsia appears to be slower than VA, with a timescale of up to 1 year.^31^ It may be necessary for the network to learn additional biomarkers, as observed in eyes with ERM, where a higher central retinal thickness correlated with postoperative VA but deep retinal folds correlated with postoperative metamorphopsia.^32^

In conclusion, the deep learning–based neural network demonstrated good accuracy in correctly predicting improvement in visual outcome in approximately three-quarters of patients, with good sensitivity and reasonable specificity. This machine learning–based personalized therapeutic strategy for ERM management could contribute to better patient counseling and decision-making. Further studies are required to assess the clinical potential and to improve the accuracy with a larger number of cases.
